# Text-mined dataset of solid-state syntheses with impurity phases using Large Language Model

**DOI:** 10.1038/s41597-025-06222-y

**Published:** 2025-12-16

**Authors:** Sanghoon Lee, Kevin Cruse, Viktoriia Baibakova, Gerbrand Ceder, Anubhav Jain

**Affiliations:** 1 Lawrence Berkeley National Laboratory, Energy Technologies Area, Berkeley, USA; 2 University of California, Berkeley, Department of Materials Science and Engineering, Berkeley, USA; 3 Lawrence Berkeley National Laboratory, Materials Sciences Division, Berkeley, USA

**Keywords:** Materials science, Cheminformatics

## Abstract

Solid-state synthesis is widely used to obtain various inorganic materials, such as battery materials and bulk thermoelectrics. Despite its prevalence, the process remains challenging due to the lack of a general theory and well-understood underlying reaction mechanisms. While prior works have successfully extracted structured datasets from literature, they often neglect product phase purity or yield. In this work, we construct a solid-state synthesis dataset consisting of 80,806 syntheses extracted with a large language model (LLM), including 18,869 reactions with impurity phase(s). Our dataset not only validates expected thermodynamic trends for impurity phase formation but also identifies challenging cases where impurity phases emerge even when the target phase is significantly more stable.

## Background & Summary

Solid-state synthesis is widely used to produce various targets including battery materials and nanomaterials^1^. Despite its widespread application, theories for synthesis are still developing and are not yet fully unified under a general framework^2,3^. The formation of a compound typically requires an interplay of thermodynamic driving force, diffusion, and other transport phenomena across multiple interfaces^3–5^.

In the quest for improved understanding and control of these multi-step, multi-variable reactions, data-driven strategies have gained considerable momentum. The quantity and quality of available data can be the deciding factor in the success of machine learning (ML) models^6^. Crystal structure and energy representation have benefitted tremendously from the development of both experiment-based^7^ and computation-based^8^ databases. While these databases serve as invaluable resources, providing large volumes of structured materials data, their coverage of synthesis details remains limited.

Recent advances in ML offer promising avenues for tackling the complexities of solid-state synthesis. In particular, learned representations (embeddings) of materials from the literature have led to new pathways for materials discovery^9–11^. Progress in machine-learned interatomic potentials^12^ has further enabled highly accurate energy calculations of crystal structures, with some models demonstrating the ability to generate structures^13,14^ that are experimentally realized via high-throughput autonomous synthesis^15–17^.

Previous studies have successfully derived structured datasets from existing literature. Extracting structured datasets from existing literature offers distinct advantages over other databases due to its expansive and customizable scope. Kononova *et al*. have extracted a dataset of 19,488 solid-state syntheses^18^. This motivated several studies including target stoichiometry prediction^19^, reaction pathway predictions^20,21^, precursor and synthesis condition recommendations^22–24^. Building on the broader interest in synthesis, Wang et al. compiled a large dataset of solution-based reactions^25^. Although literature-based datasets have been noted to exhibit human-driven bias^26,27^, these datasets have proven highly valuable as consolidated experimental datasets, serving as effective validation sets for theory-based prediction models^20,21^.

However, one major bottleneck in applying data-driven methods to real-world syntheses is the lack of negative data - i.e., records of “failed” or phase-impure experiments. In the literature, such syntheses are rarely highlighted, which can introduce bias into our understanding of reaction mechanisms and limit the predictive power of ML models^28^. While truly “failed” reactions are rarely reported, we focus in this paper on reactions that produce undesired byproducts or “impurity phases” (also referred to as “secondary phases” or “minor phases”) along with the target phase in solid-state synthesis, as these minor phases can contain valuable information of the reaction pathway that was taken.

Minor deviations in synthesis conditions can trigger the formation of impurity phases during inorganic syntheses. As an example, in the synthesis of the multiferroic BiFeO_3_, its narrow thermodynamic stability range and the volatility of bismuth frequently result in the formation of parasitic phases of Bi_2_Fe_4_O_9_ and Bi_25_FeO_40_ as impurity phases^29–32^. Similarly, the fabrication of the thin-film Cu_2_ZnSnS_4_ often leads to competing phases including ZnS, Cu_2_S, SnS_2_, and Cu_2_SnS_3_ which are known to be detrimental to its device performance^33,34^.

Recently, Thway *et al*. presented a case study of 82 ternary chalcogenide syntheses, incorporating phase purity and leveraging ChatGPT to process 168 relevant papers^35^. This work also trained a decision tree classifier using a dataset of 168 papers with 61.5% test accuracy on the formation of impurity phases, highlighting the scientific possibilities of such datasets. In this work, we construct a structured solid-state synthesis dataset of 80,806 solid-state syntheses, featuring 18,869 reactions that explicitly report impurity phase(s). This approach aims to fill the gap left by conventional literature mining efforts and further pave the way for more robust data-driven approaches in elucidating solid-state synthesis routes.

## Methods

**Fig. 1:**
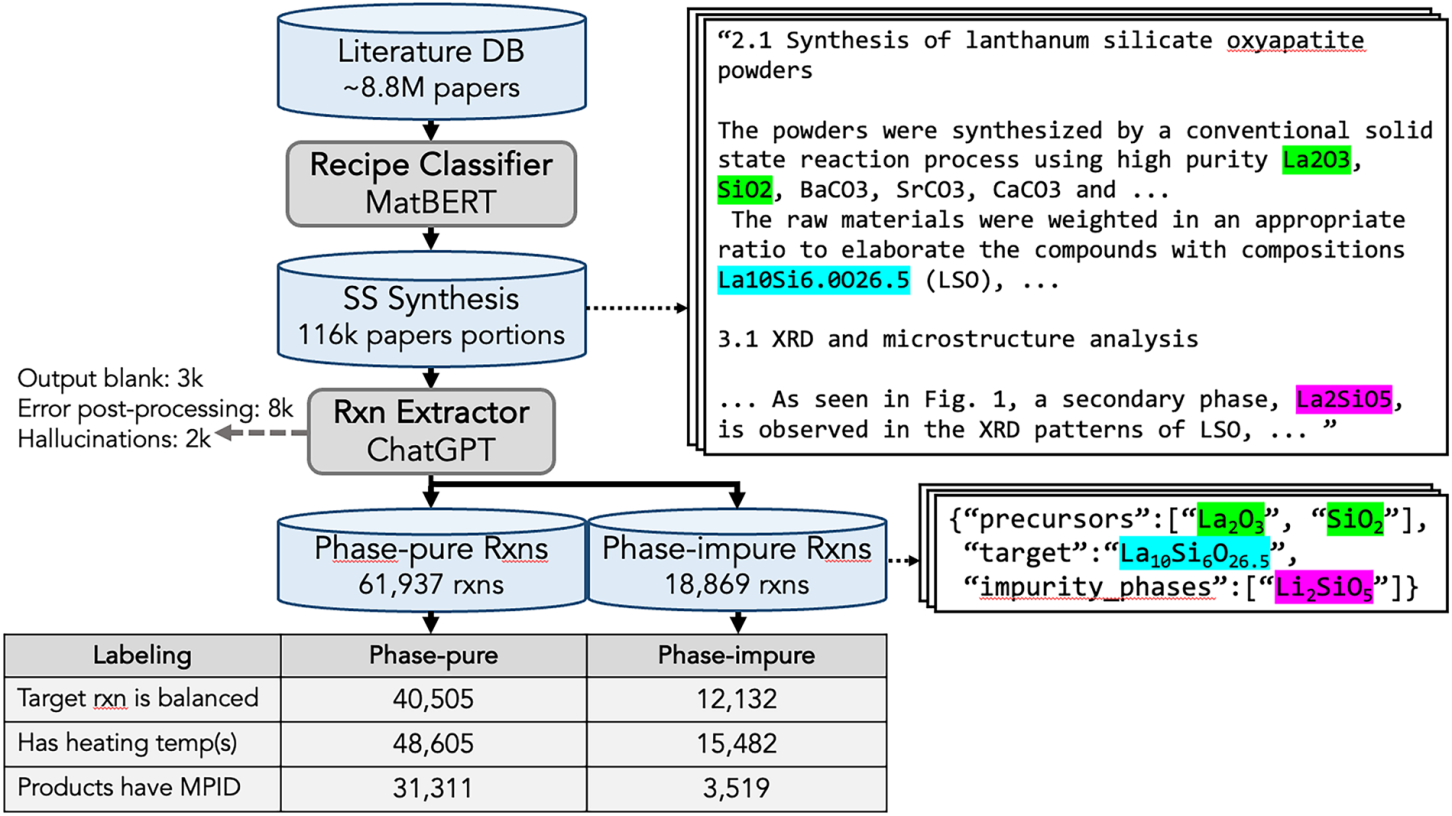
Schematics of solid-state synthesis extraction pipeline. The right panel shows an example of processing a recipe paragraph using simplified ChatGPT prompts. The paper portion consists of the abstract, solid-state synthesis paragraph(s), results, and discussion sections. Products refer to the target phase and, if present, any impurity phases. The full descriptions of the prompts are in Supplementary Information. The example paper portion is adopted from XG Cao *et al*., J. Mater. Chem. A, 2, 20739-20747 (2014)^67^.

The recipe extraction pipeline is described in Fig. 1. The final dataset includes 80,806 reported solid-state synthesis reactions, 18,869 with impurity phases, and 61,937 without reported impurity phase. For enhanced precision, we excluded publications using multiple synthesis protocols.

### Content Acquisition

We started from a literature database of 8.4 million publications sourced from the following publishers (retrieved as XML files), in a similar fashion to the methods in Kononova *et al*.^18^:

- American Chemical Society (2000–2020)
- American Institute of Physics (2000–2023)
- American Physical Society (2000–2020)
- Elsevier (2000–2023)
- Institute of Physics (2000–2024)
- Springer Nature (2000–2024)
- The Royal Society of Chemistry (2000–2023)
- Wiley (2000–2020)

For publishers with broader topic distributions (e.g., Elsevier) we manually confined our initial search for papers related to journals related to materials science, engineering, physics, chemistry, and other topics that would cover inorganic synthesis. Papers were gathered via dedicated publisher API, secure file transfer protocol (SFTP), or direct web scraping with Scrapy-based tools (https://www.scrapy.org/). We only gathered papers from 2000 and after since those are commonly provided as HTML/XML and parsing PDFs via OCR is difficult for scientific papers. The articles were parsed into a machine readable format using the Beautiful Soup package (https://beautiful-soup-4.readthedocs.io/en/latest/). The papers and their parsed contents are stored across internally maintained MongoDB collections (https://www.mongodb.com/). GitHub repositories are available containing relevant code for scraping (https://github.com/kevcruse96/Borges) and parsing (https://github.com/kevcruse96/LimeSoup) according to each publisher. We also share the DOI list of all the publications in the literature database (https://github.com/slee-lab/solid-state-recipes-with-impurity).

### Recipe Paragraph Classification

We collected solid-state synthesis paragraphs using a machine-learned paragraph classifier. Based on our previous work^18^, we manually determined a list of keywords that appear in the section headings of synthesis paragraphs (“preparation”, “prepared”, “experiment”, “method”, “material”, “synthes”). To save computing resources, we designed a regular-expression-based filter to screen for synthesis paragraphs using these keywords. Out of a total of 292M paragraphs, we filtered out 233.5M as non-synthesis. We then applied a MatBERT^10^ classifier to the remaining 58.5M paragraphs, classifying each (using a batch size of 16) into one of the following five classes: solid-state, sol-gel, precipitation, hydrothermal, and something else. MatBERT identified 145K solid-state synthesis paragraphs; we then aggregated at the paper level, selecting papers containing one or more solid-state synthesis paragraphs and no paragraphs labeled as other classes. We applied an additional filtering step to remove paragraphs with scores below 0.8, thereby improving the reliability of the extracted data.

### Recipe Extraction and Labeling

For each solid-state synthesis paper, only relevant portions (abstract, synthesis paragraphs, results and discussions) are collected using the section headings. Introduction and conclusion sections are discarded as they often involve repeated information or discuss synthesis processes and results of the previous works.

Next, the reactions (precursors, targets, and impurity phases) are extracted using few-shot LLM^36,37^(gpt-4o-2024-05-13 with temperature = 0) using OpenAI API. The example input and output are demonstrated in Fig. 1. The full prompts and configuration used are described in Supplementary Information. The validation of the reaction extractor is described in Technical Validation.

The dataset is then post-processed and labeled with additional features. The raw LLM output string gets parsed to detect target, precursors and impurity phases, and duplicate reactions occurring within the same output (i.e., from one paper portion) are removed. Firstly, the materials were detected from the output string using MaterialParser, and reactions were balanced using ReactionBalancer^18^ consistent with Kononova *et al*. We introduced an additional filter for MaterialParser material detection by using LLM as a verifier for cases where the input string was different from the detected material formula (described further in Supplementary Information). An optional feature of mp_id was added by querying materials (target and impurity phases) formulas to the Materials Project (MP) and fetching mp_id of the most stable entry. Also, reaction conditions including mixing and heating conditions are extracted using synthesis action retriever^38^.

## Data Records

The dataset of 80,806 solid-state synthesis reactions is provided as a JSON file available at 10.6084/m9.figshare.30423274^39^. The data record format and content are described in Table 1. Each record corresponds to a solid-state synthesis reaction extracted from a publication with a DOI of the source publication. A reaction is described by the target reaction and impurity phase reaction(s), along with each material entity and synthesis operations. An example entry from the dataset is shown in Supplementary Information.

**Table 1:** Data record format.

Data Description	Data Key Label	Data Type
DOI of the original paper	DOI	string
Balanced target reaction	target_reaction	if not balanced, blank list; if balanced, list of dicts:
		- left: list of {material: string, amount: float}
		- right: list of {material: string, amount: float}
Balanced side reactions for impurity phase	impurity_phase_reaction	list of dicts, same as target_reaction
Target material	target	list of dicts:
		- material_string: string
		- material_formula: string or *None*
		- composition: list of {formula: string, elements: {element: amount of element}, amount: string}
		- additives: list of strings
		- element_vars: {var: list of strings}
		- amounts_vars: {var: list of dicts}
		- oxygen_deficiency: boolean
		- mp_id: string
Impurity phase	impurity_phase	list of dicts (See target)
Precursor materials	precursors	list of dicts (See target)
Synthesis conditions extracted from the paper	conditions_forDOI	list of dicts:
		- token: string
		- type: string
		- heating_temperature: {value: float, unit: string}
		- heating_time: {value: float, unit: string}
		- heating_atmosphere: list of strings
		- mixing_device: list of strings
		- mixing_media: list of strings

## Data Overview

The top 8 common reactions and corresponding impurity phases in the phase-impure dataset are summarized in Table 2. BiFeO_3_ (BFO), a room-temperature multiferroic, is challenging to synthesize as a single phase^29–32,40^ due to formation of Bi_2_Fe_4_O_9_ and Bi_25_FeO_39_^41^ at elevated temperatures, and the formation of such phases is also known to be sensitive to synthesis conditions including (precursor) impurities^42^. In our dataset, BFO is the most prevalent target in the phase-impure syntheses, while interestingly, it is also frequently present in the phase-pure dataset.

**Table 2:** Most common reactions with impurity phases.

Reactions (Fraction pure)	Impurity phases
0.5 Bi_2_O_3_ + 0.5Fe_2_O_3_ → BiFeO_3_ (107 / 221)	Bi_2_5FeO_40_ + Bi_2_Fe_4_O_9_(18)
	unspecified(18)
	Bi_2_Fe_4_O_9_(17)
	Bi_2_5FeO_39_ + Bi_2_Fe_4_O_9_(16)
	Bi_25_FeO_40_(6)
CaCO_3_ + 3CuO + 4TiO_2_ → CaCu_3_Ti_4_O_12_ + CO_2_(119/179)	unspecified (15)
	CaTiO_3_ + CuO(7)
	Cu_2_O + CuO(5)
2BaCO_3_ + 3CuO + 0.25O_2_ + 0.5Y_2_O_3_ → YBa_2_Cu_3_O_7_ + 2CO_2_(91/138)	unspecified(22)
	Y_2_BaCuO_5_(8)
2C + *Si* + 3*Ti* → Ti_3_SiC_2_ (26 / 71)	TiC(16)
	SiC + TiC(6)
	unspecified(6)
BaCO_3_ + TiO_2_ → BaTiO_3_ + CO_2_ (258 / 302)	Ba_2_TiO_4_(9)
	unspecified(6)
Al + 2*C* + 3*Ti* → Ti_3_AlC_2_ (52 / 91)	(14)
	unspecified(9)
Al_2_O_3_ + MgO → MgAl_2_O_4_ (83 / 121)	(21)
2Li_2_CO_3_ + 5TiO_2_ → Li_4_Ti_5_O_12_ + 2CO_2_ (117 / 146)	TiO_2_(12)
	unspecified(8)
	Li_2_TiO_3_ + TiO_2_(5)

CaCu_3_Ti_4_O_12_ (CCTO), was recognized in 2000 for its “giant” dielectric constant, making it a desirable candidate for capacitor applications^43,44^. Such large permittivity is widely attributed to be from a grain boundary internal barrier layer capacitance (IBLC)^45^, which is sensitive to microstructural features such as grain size^46^. However, the synthesis of single crystal CCTO is often challenging due to the appearance of tenorite (CuO) as an impurity phase, which has been reported in both stoichiometric and Cu-deficient conditions^47^. Interestingly, Marchin *et al*. found in their coprecipitation synthesis that an excess CuO in the calcined powder led to increased grain size upon sintering -ultimately enhancing the effective permittivity^48^.

Another notable material, YBa_2_Cu_3_O_*x*_ (YBCO) is a high-temperature superconductor, discovered in 1987 and synthesized through a solid-state method with heating at 930 ° C for 1 day^49,50^. However, phase-pure synthesis of YBCO via conventional solid-state synthesis is known to be challenging due to slow diffusion and potential impurity phases^51,52^.

Ti_3_SiC_2_, Ti_3_AlC_2_ and Cr_2_AlC are MAX phases that exhibit both metallic and ceramic properties^53^. These phases demonstrate high electrical and thermal conductivity, along with excellent corrosion resistance. The synthesis reactions of Ti_3_AlC_2_ in our dataset highlights the crucial role of precursor selection in determining phase purity. When using elemental precursors Al + C + Ti, Ti_3_AlC_2_ was synthesized both phase-pure (52 data points) and phase-impure (39), with 4 data points that form TiC + Ti_2_AlC impurity phases (see Table S3). This suggests that while the elemental route is viable, impurity phase formation is likely. In contrast, starting with the compound precursors of Ti_2_AlC + TiC markedly favors the production of phase-pure Ti_3_AlC_2_, observed in 32 phase-pure data points. Although there are 2 phase-impure data points using this route, these had impurity phases of only unreacted TiC precursor. The predominance of pure phases with compound precursors implies that pre-formed carbides create a more controlled synthesis pathway, effectively minimizing the formation of additional phases.

Interestingly, we observed a considerable amount of cases where the impurity phases consisted of unreacted precursors. Li_4_Ti_5_O_12_ spinel is a battery material used both as cathode and anode material^54^, and 14 out of 29 datapoints in the phase-impure dataset had TiO_2_ as one of the impurity phases. Documenting these unreacted precursors is particularly valuable, as it can easily be an indicator of whether a given reaction has reached completion under the reported conditions. For instance, the presence of unreacted precursors can pinpoint specific bottlenecks in reaction kinetics, such as insufficient diffusion or driving force that hinders a synthesis pathway. Beyond serving as an indicator, these synthesis data points can guide subsequent optimization efforts for autonomous labs.

We further investigated reactions in which impurity phases include unreacted precursors more frequently. Table S4 shows reactions with the fraction of reactions with unreacted precursors higher than 0.39, and Table S5 shows precursors that are more or less likely to appear as impurity phases. TiC, for example, is a prominent precursor that is detected as an impurity phase in a majority of phase-impure data points of Al + Ti + 2TiC → Ti_3_AlC_2_ and Si+Ti+2TiC → Ti_3_SiC_2_.

## Technical Validation

### Validation of Recipe Paragraph Classifier

To evaluate the performance of the paragraph classification pipeline, we constructed a stratified validation dataset consisting of 480 paragraphs with manually evaluated class labels. We randomly sampled 10 paragraphs for each of 8 publishers across 6 classes: solid-state, sol-gel, hydrothermal, precipitation, something else (determined by MatBERT), and filtered out (determined by a keyword filter). The dataset was balanced by publisher and class label. Two materials science PhD candidates then independently annotated the sampled paragraphs, agreeing on the labels for 429 out of 480 paragraphs (89%). The ground truth for the class labels was subsequently determined through discussion of the annotators until an agreement was reached. Human annotator 1 matched the ground truth for 446 paragraphs (93%), human annotator 2 for 459 paragraphs (96%), and the MatBERT classifier matched the ground truth for 412 paragraphs (86%). Finally, for all 6 classes, we built a confusion matrix comparing the ground truth with the classifier predictions to calculate recall and precision. MatBERT achieved a precision of 0.84 and a recall of 0.97 for solid-state synthesis classification, as shown in Table 3. Further details on the validation and the confusion matrix (Figure S1) are provided in the Supplementary Information.

**Table 3:** Evaluation metrics of recipe extraction. For reaction extraction (target, precursors, and impurity phases), a validation set was randomly pulled from 116K SS synthesis papers and annotated.

Entity	Method	Precision	Recall	F1 score	Support (TP+FN)
Recipe Classifier: solid-state synthesis	MatBERT	0.84	0.97	0.90	80
Targets	GPT4o + post-process	0.90	0.69	0.78	83
Precursors	GPT4o + post-process	0.95	0.97	0.96	179
Impurity phases	GPT4o + post-process	0.86	0.90	0.88	21

### Validation of Reaction Extractor

To assess the accuracy of the reaction extraction pipeline, we first randomly sampled 78 paper portions from 116K solid-state synthesis papers. These were manually annotated, yielding a total of 98 reactions from 59 paper portions, with each reaction containing a target, precursors, and impurity phases if reported. The remaining 19 paper portions were excluded from annotation due to various challenges to provide straightforward ground truth annotation using the established annotation schema. These include cases with ambiguous synthesis types or targets (e.g. glass synthesis), and cases requiring figures^55^ or tables^56^ to identify synthesis target composition or impurity phase formation.

The validation dataset was then processed through the same recipe extraction and post-processing pipeline as the main dataset, as outlined in Fig. 1. During this step, one paper portion was removed due to a blank LLM output, and four others were excluded due to invalid GPT-4o output formats (e.g. missing closing bracket) hence filtered out in post-processing. After this screening process, the final validation dataset consisted of 83 reactions from 54 paper portions.

To evaluate extraction performance, we computed micro-averaged precision, recall, and F1 scores of the final validation dataset for three reaction entity types: target materials, precursors, and impurity phases. Ground truth annotations were first matched to extracted reactions based on target material formulas. If an annotated paper portion contained three reactions with targets A, B, and C, but the extraction produced two reactions with targets A and D, this was recorded as one true positive (A), one false positive (D), and two false negatives (B, C).

Once reactions were matched, extracted precursors and impurity phases were compared against their ground-truth annotations. For example, in a reaction extracted from Kim *et al*.^57^, the model identified the target material of '(1-x)NdAlO3-xCaTiO3', with precursors ['Nd2O3', 'Al2O3', 'CaCO3', 'TiO2'], and impurity phases as ['Nd4Al2O9', 'Al-rich phase']. The ground-truth annotation for this reaction listed the same target and precursors but included only ['Nd4Al2O9'] as an impurity phase. Based on this comparison, the precursor extraction records four true positives, while impurity phase extraction records one true positive and one false positive. These counts were aggregated across all reactions to compute micro-averaged precision, recall, and F1 scores, as shown in Table 3.

Overall, our extraction pipeline achieved F1 scores above 0.78; metrics can further increase when filtering by labels - for instance, impurity phase F1 increases from 0.88 to 0.93 when using only balanced reactions. Despite the inherent complexity of capturing long-range context-dependent features, these results underscore the reliability of our approach in straightforward scenarios.

The few-shot GPT-4o achieved high precision (0.86–0.95) overall, despite frequent challenges such as long-range linking, since impurity phase information is often reported in the discussion section rather than within the recipe itself. We also observed promising cases where the model correctly captured negative or preventive statements, for example: “procedure A was conducted to prevent phase B formation” and “phases C and D are commonly known to form...”. These instances indicate that large language models can indeed recognize and extract deeper contextual clues about undesired phases and their mitigation strategies.

### Coverage of the dataset

**Fig. 2:**
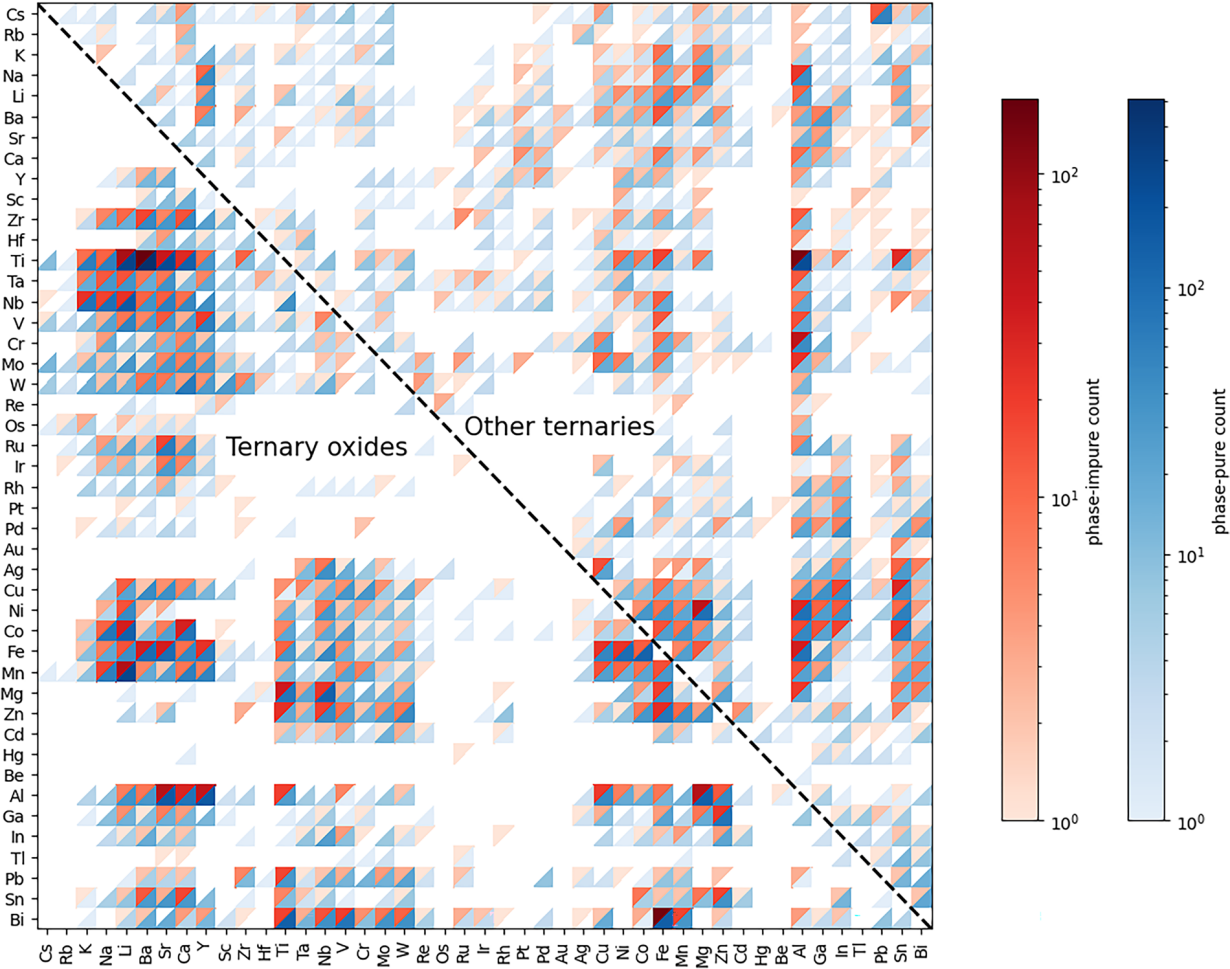
Ternary compound targets in the phase-impure/pure dataset. The lower left triangle shows oxides, while the upper right triangle includes ternary compounds where only two of the elements in the fixed elements set (shown in the axis) are present, excluding those containing all three.

Figure 2 shows the coverage of the dataset in ternary compound target systems. For oxides, there is a clear cluster of high-density data (both red and blue) in the transition metal (Ti, V, Cr, Mn, Fe, Co, Ni) and alkali and alkaline earth elements, suggesting these are among the most extensively studied and synthesized ternary systems. This is expected given their technological importance in areas such as batteries and catalysis.

Overall, the presence of both phase-pure (blue in Fig. 2) and phase-impure (red) in most cells suggests that achieving phase purity can be challenging even in well-studied systems. As an example, the La-Ti-O system has 47 phase-pure and 12 phase-impure reactions. While the reaction La_2_O_3_ + 2TiO_2_ → La_2_Ti_2_O_7_ is mostly phase pure, with 96% of reactions yielding a pure phase (Table S1), the 12 phase-impure reactions consist of cases involving alternative precursors, such as ${{\rm{La}}}_{2}{({{\rm{CO}}}_{3})}_{3}$, or different target compounds, such as La_2_TiO_5_. On the other hand, some systems such as Y-Nb-O and Mo-Cs-O have no phase-impure reactions but only phase-pure reactions (39 and 12 reactions respectively), indicating better synthetic control.

### Thermodynamic driving force for impurity phase formation

Thermodynamic analyses often rely on the concept of energy above hull (*E*_hull_) to assess the synthesizability of a compound. A material with zero energy above hull is predicted to be stable, while values slightly above zero typically indicate metastable compounds that may still be experimentally synthesizable, though this correlation has been shown to be necessary but not sufficient to guarantee possible synthesis^58^. The *E*_hull_ has proven useful in high-throughput computational materials design. However, the accuracy of first-principles energy calculations can be limited by factors such as exchange-correlation functionals (e.g. GGA^59^, r^2^SCAN^60^) and temperature-dependent entropy corrections^61^.

**Fig. 3:**
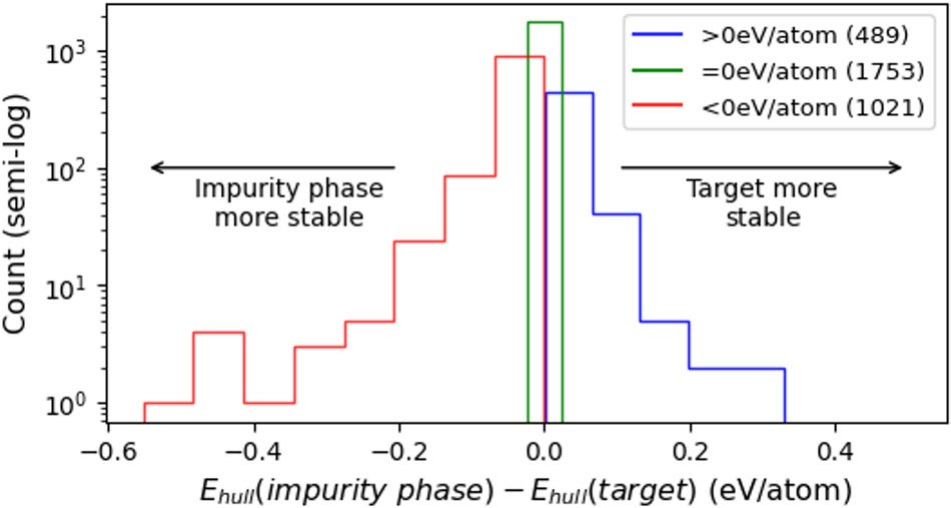
Histogram of difference in energies above hulls in target-impurity pairs in phase-impure synthesis dataset (log-scaled). Target and impurity phase material formulas were queried to Materials Project to fetch energy above hull. Four outliers were manually checked and filtered out.

To verify the quality of the phase-impure reactions data, we collected 6,542 reactions that have specified impurity phase and balanced target reactions, from 18,869 phase-impure syntheses. Then, we screened for 3,267 reactions in which all materials (i.e. precursors, target, and any reported impurity phases) could be mapped to entries in the Materials Project (MP) database with GGA/GGA+U calculations^8^. Figure 3 shows the distribution of *E*_hull_ of the target phase and the impurity phase for those 3,267 reactions. In roughly 31% of pairs, the impurity phase has lower *E*_hull_ than the target, implying a straightforward thermodynamic explanation for why impurity phases formed. However, 15% of the reactions show the impurity phase having a higher *E*_hull_ than the target, yet it still appears experimentally. Moreover, 1,752 reactions have both target and impurity phases on the convex hull (*E*_hull_ = 0), underscoring the limitation of relying solely on *E*_hull_ to predict reaction outcomes. Further analysis beyond *E*_hull_ is in Supplementary Information (Figure S2).

The utility of the literature-mined dataset must be considered in light of two fundamental challenges. First, as Raccuglia *et al*. demonstrated, published literature typically omits failed experiments, creating a systematic bias toward successful outcomes^28^. These “dark reactions" of unsuccessful attempts, though valuable for understanding synthesis parameters, often remain buried. Second, as highlighted in Jia *et al*., researchers tend to explore confined regions of synthesis space rather than sampling randomly, leading to potentially overlooked opportunities in unexplored parameter regions^26^.

Our dataset of 80,806 solid-state syntheses, including 18,869 with reported impurity phases, provides unique value in addressing these challenges. The inclusion of phase-impure syntheses is particularly significant as “failed” phase-pure syntheses. This allows immediate cross-validation of any given synthesis recipe against multiple reported attempts, including both successful and unsuccessful outcomes, providing a more complete picture of synthesis reproducibility.

This dataset enables three key applications. First, when multiple groups report consistent phase-pure outcomes for a particular synthetic route (as shown in Table S1), researchers can confidently “exploit” these reproducible recipes and extract general principles for successful synthesis. Second, when different groups report conflicting outcomes under seemingly identical conditions (as shown in some examples like BFO in Table 2), it suggests the presence of critical but hidden variables that necessitate further investigation. These discrepancies can guide focused studies to identify and understand undocumented factors affecting phase purity. Third, by mapping the documented synthesis space, our dataset reveals regions where exploration has been limited by anthropogenic bias. Also shown in an example of gold nanoparticle synthesis^62^, these gaps represent opportunities for systematic investigation beyond conventional parameter ranges, potentially leading to the discovery of rarely used precursor sets, or added protocols in mixing or heating, ultimately for improved understanding of reaction mechanisms. This comprehensive view of both explored and unexplored synthesis space, combined with phase purity information, provides a foundation for more systematic and efficient materials synthesis development.

## Supplementary information

Supplementary Information for Text-mined dataset of solid-state syntheses with impurity phases using Large Language Model

## Data Availability

The dataset is in a single JSON file that can be accessed in programming languages including Python. It is publicly available at 10.6084/m9.figshare.30423274^39^.

## Code availability

The code used to collect and process the dataset is available at https://github.com/CederGroupHub/text-mined-synthesis_public^18^. Additionally, the project used OpenAI API for inference using gpt-4o-2024-05-13, Qwen 2.5^63^ using LBNL CBORG API (https://cborg.lbl.gov/) and other open libraries including pymatgen, mp-api, numpy, matplotlib, Synthesis Paragraph Classifier https://github.com/CederGroupHub/MatBERT-synthesis-classifier^64^, Synthesis Action Retrieval 10.5281/zenodo.6383380^65^. The code used for post-processing LLM outputs and a tutorial are available at https://github.com/slee-lab/solid-state-recipes-with-impurity^66^.

## References

[1] Kumar, A. *et al*. Solid-state reaction synthesis of nanoscale materials: Strategies and applications. *Chemical Reviews* **122**, 12748–12863, 10.1021/acs.chemrev.1c00637 (2022). 35715344 10.1021/acs.chemrev.1c00637

[2] Kohlmann, H. Looking into the black box of solid-state synthesis. *European Journal of Inorganic Chemistry* **2019**, 4174–4180, 10.1002/ejic.201900733 (2019).

[3] Aykol, M., Montoya, J. H. & HummelshØj, J. Rational solid-state synthesis routes for inorganic materials. *Journal of the American Chemical Society* **143**, 9244–9259, 10.1021/jacs.1c04888 (2021). 34114812 10.1021/jacs.1c04888

[4] Chamorro, J. R. & McQueen, T. M. Progress toward solid state synthesis by design. *Accounts of Chemical Research* **51**, 2918–2925, 10.1021/acs.accounts.8b00382 (2018). 30299082 10.1021/acs.accounts.8b00382

[5] Jansen, M. A concept for synthesis planning in solid-state chemistry. *Angewandte Chemie International Edition* **41**, 3746–3766 (2002). 12386844 10.1002/1521-3773(20021018)41:20<3746::AID-ANIE3746>3.0.CO;2-2

[6] Kim, E., Huang, K., Jegelka, S. & Olivetti, E. Virtual screening of inorganic materials synthesis parameters with deep learning. *npj Computational Materials* **3**, 10.1038/s41524-017-0055-6 (2017).

[7] Zagorac, D., Müller, H., Ruehl, S., Zagorac, J. & Rehme, S. Recent developments in the Inorganic Crystal Structure Database: theoretical crystal structure data and related features. *Journal of Applied Crystallography* **52**, 918–925, 10.1107/S160057671900997X (2019). 31636516 10.1107/S160057671900997X PMC6782081

[8] Jain, A. *et al*. Commentary: The Materials Project: A materials genome approach to accelerating materials innovation. *APL Materials* **1**, 011002, 10.1063/1.4812323 (2013).

[9] Tshitoyan, V. *et al*. Unsupervised word embeddings capture latent knowledge from materials science literature. *Nature* **571**, 95–98, 10.1038/s41586-019-1335-8 (2019). 31270483 10.1038/s41586-019-1335-8

[10] Trewartha, A. *et al*. Quantifying the advantage of domain-specific pre-training on named entity recognition tasks in materials science. *Patterns* **3**, 10.1016/j.patter.2022.100488 (2022). 10.1016/j.patter.2022.100488 PMC9024010 35465225

[11] Qu, J. *et al*. Leveraging language representation for materials exploration and discovery. *npj Computational Materials* **10**, 58, 10.1038/s41524-024-01231-8 (2024).

[12] Xie, T. & Grossman, J. C. Crystal graph convolutional neural networks for an accurate and interpretable prediction of material properties. *Phys. Rev. Lett.* **120**, 145301, 10.1103/PhysRevLett.120.145301 (2018). 29694125 10.1103/PhysRevLett.120.145301

[13] Merchant, A. *et al*. Scaling deep learning for materials discovery. *Nature* **624**, 80–85, 10.1038/s41586-023-06735-9 (2023). 38030720 10.1038/s41586-023-06735-9 PMC10700131

[14] Zeni, C. *et al*. A generative model for inorganic materials design. *Nature*10.1038/s41586-025-08628-5 (2025). 10.1038/s41586-025-08628-5 PMC11922738 39821164

[15] Szymanski, N. J. *et al*. An autonomous laboratory for the accelerated synthesis of novel materials. *Nature* **624**, 86–91, 10.1038/s41586-023-06734-w (2023). 38030721 10.1038/s41586-023-06734-w PMC10700133

[16] Jiang, Y. *et al*. An artificial intelligence enabled chemical synthesis robot for exploration and optimization of nanomaterials. *Science Advances* **8**, eabo2626, 10.1126/sciadv.abo2626 (2022). 36206340 10.1126/sciadv.abo2626 PMC9544322

[17] Tao, H. *et al*. Self-driving platform for metal nanoparticle synthesis: Combining microfluidics and machine learning. *Advanced Functional Materials* **31**, 2106725, 10.1002/adfm.202106725 (2021).

[18] Kononova, O. *et al*. Text-mined dataset of inorganic materials synthesis recipes. *Scientific Data* **6**, 10.1038/s41597-019-0224-1 (2019). 10.1038/s41597-019-0224-1 PMC6794279 31615989

[19] Malik, S. A., Goodall, R. E. & Lee, A. A. Predicting the outcomes of material syntheses with deep learning. *Chemistry of Materials* **33**, 616–624, 10.1021/acs.chemmater.0c03885 (2021).

[20] McDermott, M. J., Dwaraknath, S. S. & Persson, K. A. A graph-based network for predicting chemical reaction pathways in solid-state materials synthesis. *Nature Communications* **12**, 3097, 10.1038/s41467-021-23339-x (2021). 34035255 10.1038/s41467-021-23339-x PMC8149458

[21] McDermott, M. J. *et al*. Assessing thermodynamic selectivity of solid-state reactions for the predictive synthesis of inorganic materials. *ACS Central Science* **9**, 1957–1975, 10.1021/acscentsci.3c01051 (2023). 37901171 10.1021/acscentsci.3c01051 PMC10604012

[22] He, T. *et al*. Similarity of precursors in solid-state synthesis as text-mined from scientific literature. *Chemistry of Materials* **32**, 7861–7873, 10.1021/acs.chemmater.0c02553 (2020).

[23] Huo, H. *et al*. Machine-learning rationalization and prediction of solid-state synthesis conditions. *Chemistry of Materials* **34**, 7323–7336, 10.1021/acs.chemmater.2c01293 (2022). 36032555 10.1021/acs.chemmater.2c01293 PMC9407029

[24] Prein, T. *et al*. Reaction graph networks for inorganic synthesis condition prediction of solid state materials. In *AI for Accelerated Materials Design - NeurIPS 2024* (2024).

[25] Wang, Z. *et al*. Dataset of solution-based inorganic materials synthesis procedures extracted from the scientific literature. *Scientific Data* **9**, 231, 10.1038/s41597-022-01317-2 (2022). 35614129 10.1038/s41597-022-01317-2 PMC9132903

[26] Jia, X. *et al*. Anthropogenic biases in chemical reaction data hinder exploratory inorganic synthesis. *Nature* **573**, 251–255, 10.1038/s41586-019-1540-5 (2019). 31511682 10.1038/s41586-019-1540-5

[27] Sun, W. & David, N. A critical reflection on attempts to machine-learn materials synthesis insights from text-mined literature recipes. *Faraday Discuss.* **256**, 614–638, 10.1039/D4FD00112E (2025). 39351769 10.1039/d4fd00112e

[28] Raccuglia, P. *et al*. Machine-learning-assisted materials discovery using failed experiments. *Nature* **533**, 73–76, 10.1038/nature17439 (2016). 27147027 10.1038/nature17439

[29] Makridis, A., Myrovali, E., Sakellari, D. & Angelakeris, M. Tuning the structural and the magnetic properties of bifeo3 magnetic nanoparticles. *physica status solidi (b)* **257**, 2000005, 10.1002/pssb.202000005 (2020).

[30] Andryushin, K. P. *et al*. Reasons for the high electrical conductivity of bismuth ferrite and ways to minimize it. *Applied Sciences* **11**, 10.3390/app11031025 (2021).

[31] Kirsch, A. *et al*. Control of crystallization pathways in the bifeo3-bi2fe4o9 system. *Chemistry of Materials* **37**, 338–348, 10.1021/acs.chemmater.4c02656 (2025). 39830220 10.1021/acs.chemmater.4c02656 PMC11736678

[32] Wesley, C. *et al*. Solid state synthesis of bifeo3 occurs through the intermediate bi25feo39 compound. *Journal of the American Ceramic Society* **107**, 3716–3723, 10.1111/jace.19702 (2024).

[33] Wang, W. *et al*. The effects of sns2 secondary phases on cu2znsns4 solar cells: a promising mechanical exfoliation method for its removal. *J. Mater. Chem. A* **6**, 2995–3004, 10.1039/C7TA08242H (2018).

[34] Kumar, M., Dubey, A., Adhikari, N., Venkatesan, S. & Qiao, Q. Strategic review of secondary phases, defects and defect-complexes in kesterite czts-se solar cells. *Energy Environ. Sci.* **8**, 3134–3159, 10.1039/C5EE02153G (2015).

[35] Thway, M. *et al*. Harnessing gpt-3.5 for text parsing in solid-state synthesis - case study of ternary chalcogenides. *Digital Discovery* **3**, 328–336, 10.1039/D3DD00202K (2024).

[36] Sayeed, H. M., Mohanty, T. & Sparks, T. D. Annotating materials science text: A semi-automated approach for crafting outputs with gemini pro. *Integrating Materials and Manufacturing Innovation* **13**, 445–452, 10.1007/s40192-024-00356-4 (2024).

[37] Yang, S. J. *et al*. Accurate prediction of experimental band gaps from large language model-based data extraction (2023).

[38] Wang, Z. *et al*. Ulsa: unified language of synthesis actions for the representation of inorganic synthesis protocols. *Digital Discovery* **1**, 313–324, 10.1039/D1DD00034A (2022).

[39] Lee, S. *et al*. Text-mined dataset of solid-state syntheses with impurity phases using Large Language Model, 10.6084/m9.figshare.30423274 (2025). 10.1038/s41597-025-06222-y PMC12717135 41402340

[40] Cruse, K. *et al*. Text mining the literature to inform experiments and rationalize impurity phase formation for bifeo3. *Chemistry of Materials* **36**, 772–785, 10.1021/acs.chemmater.3c02203 (2024). 38282687 10.1021/acs.chemmater.3c02203 PMC10809418

[41] Selbach, S. M., Einarsrud, M.-A. & Grande, T. On the thermodynamic stability of bifeo3. *Chemistry of Materials* **21**, 169–173, 10.1021/cm802607p (2009).

[42] Valant, M., Axelsson, A.-K. & Alford, N. Peculiarities of a solid-state synthesis of multiferroic polycrystalline bifeo3. *Chemistry of Materials* **19**, 5431–5436, 10.1021/cm071730+ (2007).

[43] Subramanian, M., Li, D., Duan, N., Reisner, B. & Sleight, A. High dielectric constant in acu3ti4o12 and acu3ti3feo12 phases. *Journal of Solid State Chemistry* **151**, 323–325, 10.1006/jssc.2000.8703 (2000).

[44] Homes, C. C., Vogt, T., Shapiro, S. M., Wakimoto, S. & Ramirez, A. P. Optical response of high-dielectric-constant perovskite-related oxide. *Science* **293**, 673–676, 10.1126/science.1061655 (2001). 11474105 10.1126/science.1061655

[45] Sinclair, D. C., Adams, T. B., Morrison, F. D. & West, A. R. Cacu3ti4o12: One-step internal barrier layer capacitor. *Applied Physics Letters* **80**, 2153–2155, 10.1063/1.1463211 (2002).

[46] Adams, T., Sinclair, D. & West, A. Giant barrier layer capacitance effects in cacu3ti4o12 ceramics. *Advanced Materials* **14**, 1321–1323 (2002).

[47] Kwon, S., Huang, C.-C., Subramanian, M. & Cann, D. P. Effects of cation stoichiometry on the dielectric properties of cacu3ti4o12. *Journal of Alloys and Compounds* **473**, 433–436, 10.1016/j.jallcom.2008.06.015 (2009).

[48] Marchin, L. *et al*. Grain growth-controlled giant permittivity in soft chemistry cacu3ti4o12 ceramics. *Journal of the American Ceramic Society* **91**, 485–489, 10.1111/j.1551-2916.2007.02174.x (2008).

[49] Wu, M. K. *et al*. Superconductivity at 93 k in a new mixed-phase y-ba-cu-o compound system at ambient pressure. *Phys. Rev. Lett.* **58**, 908–910, 10.1103/PhysRevLett.58.908 (1987). 10035069 10.1103/PhysRevLett.58.908

[50] Cava, R. J. *et al*. Bulk superconductivity at 91 k in single-phase oxygen-deficient perovskite ba_2_ycu_3_o_9-δ_. *Phys. Rev. Lett.* **58**, 1676–1679, 10.1103/PhysRevLett.58.1676 (1987). 10034505 10.1103/PhysRevLett.58.1676

[51] Pathak, L. C. & Mishra, S. K. A review on the synthesis of y-ba-cu-oxide powder. *Superconductor Science and Technology* **18**, R67, 10.1088/0953-2048/18/9/R01 (2005).

[52] Miura, A. *et al*. Observing and modeling the sequential pairwise reactions that drive solid-state ceramic synthesis. *Advanced Materials* **33**, 2100312, 10.1002/adma.202100312 (2021). 10.1002/adma.202100312 33949743

[53] Gonzalez-Julian, J. Processing of max phases: From synthesis to applications. *Journal of the American Ceramic Society* **104**, 659–690, 10.1111/jace.17544 (2021).

[54] Thackeray, M. M. & Amine, K. Li4ti5o12 spinel anodes. *Nature Energy* **6**, 683–683, 10.1038/s41560-021-00829-2 (2021).

[55] Cheng, Y. *et al*. Microstructure evolution of cr2alc max phase under a graphite bed between 1100 °C and 1500 °C. *Ceramics International* **49**, 4987–4996, 10.1016/j.ceramint.2022.10.014 (2023).

[56] Lavat, A. E. & Gayo, G. X. In situ formation of coloured m(ii)-doped zn2sio4-willemite in ceramic glazes (m=mn, co, ni, cu). *Ceramics International* **40**, 11947–11955, 10.1016/j.ceramint.2014.04.031 (2014).

[57] Kim, M.-H. *et al*. Crystal structure and microwave dielectric properties of (1-x)ndalo3-xcatio3 ceramics. *Materials Research Bulletin* **37**, 605–615, 10.1016/S0025-5408(01)00793-0 (2002).

[58] Sun, W. *et al*. The thermodynamic scale of inorganic crystalline metastability. *Science Advances* **2**, e1600225, 10.1126/sciadv.1600225 (2016). 28138514 10.1126/sciadv.1600225 PMC5262468

[59] Langreth, D. C. & Perdew, J. P. Theory of nonuniform electronic systems. i. analysis of the gradient approximation and a generalization that works. *Phys. Rev. B* **21**, 5469–5493, 10.1103/PhysRevB.21.5469 (1980).

[60] Furness, J. W., Kaplan, A. D., Ning, J., Perdew, J. P. & Sun, J. Accurate and numerically efficient r2scan meta-generalized gradient approximation. *The Journal of Physical Chemistry Letters* **11**, 8208–8215, 10.1021/acs.jpclett.0c02405 (2020). 32876454 10.1021/acs.jpclett.0c02405

[61] Bartel, C. J. *et al*. Physical descriptor for the gibbs energy of inorganic crystalline solids and temperature-dependent materials chemistry. *Nature Communications* **9**, 4168, 10.1038/s41467-018-06682-4 (2018). 30301890 10.1038/s41467-018-06682-4 PMC6177451

[62] Lee, S. *et al*. Data-driven analysis of text-mined seed-mediated syntheses of gold nanoparticles. *Digital Discovery* **4**, 93–104, 10.1039/D4DD00158C (2025).

[63] Qwen *et al*. Qwen2.5 technical report (2025).

[64] Huo, H. *et al*. Semi-supervised machine-learning classification of materials synthesis procedures. *npj Computational Materials* **5**, 1–7 (2019).

[65] kevcruse96. Cedergrouphub/synthesis-action-retriever: Second release of synthesis-action-retriever, 10.5281/zenodo.6383380 (2022).

[66] https://github.com/slee-lab/solid-state-recipes-with-impurity (2025).

[67] Cao, X. G. & Jiang, S. P. Synthesis and characterization of lanthanum silicate oxyapatites co-doped with a (a = ba, sr, and ca) and fe for solid oxide fuel cells. *J. Mater. Chem. A* **2**, 20739–20747, 10.1039/C4TA04616A (2014).

