# Supplementary material for: Text-mined dataset of solid-state syntheses with impurity phases using Large Language Model
Source: Sci Data. 2025 Dec 16;12:1969. doi: 10.1038/s41597-025-06222-y (PMC12717135; doi:10.1038/s41597-025-06222-y)
Supplement: Supplementary file 1 — Supplementary Information for Text-mined dataset of solid-state syntheses with impurity phases using Large Language Model [file 41597_2025_6222_MOESM1_ESM.pdf]

Supplementary Information for Text-mined dataset of solid-state syntheses with impurity phases using Large Language Model

1 Reaction extraction prompt

Below is the input prompt used for ChatGPT, followed by three different examples and their corresponding outputs. These examples[1–3] were selected to include diverse elements and to cover various output cases per instruction (e.g. no solid-state synthesis in input text, case with single/multiple impurity phases).

```
Your task is to identify any solid-state synthesis reactions that were performed in the
    input text into a structured format. Follow these guidelines:\n1. Identify target for
    each synthesis:\nLook for any target materials that was desired product for each
    synthesis. A reaction should always have one target material. Note that these should
    strictly be materials, preferrably with chemical formula.\n2. Identify precursors for
    each target material synthesis:\nLook for any precursor materials that was used to
    synthesize a target. A solid-state reaction should always have two or more precursor
    materials. Note that these should strictly be materials, preferrably with chemical
    formula.\n3. Identify any impurity (or secondary) phases that were formed or detected
    during each synthesis reaction. If specific impurity phases that are formed are
    presented as chemical formulas, output those chemical formulas as a bracketed list of
    those formulas. Otherwise, if impurity phase was formed but not specified, output True.
    If no impurity phase formed, output False.\n4. Structured output format:\nProvide an
    output as a list of strings, each string being one synthesis.\n\nExample output format:\
    n["<Precursor A> + <Precursor B> == <Target C> | [<impurity phase D>, <impurity phase E
    >]","<Precursor X> + <Precursor Y> + <Precursor Z> == <Target G> | True","<Precursor AA>
     + <Precursor BB> == <Target CC> | False",]
```

2 Material Formula Verifier using LLM

We used Qwen 2.5[4] to evaluate instances where `material_string` was different from `material_formula` for target and impurity phases detected by MaterialParser[5]. We observed that composite materials (e.g. `'BiFeO3-BaTiO3'` were often partially parsed by the MaterialParser. To address this, we integrated a lightweight LLM-based verification step using Qwen 2.5 to assess the correctness of the extracted formulas and ensure that full material representations were accurately identified. In case the formulas were evaluated to be incorrect, we assigned `None` for `material_formula`.

3 Example entry from the dataset


```
{’DOI’: ’10.1016/j.cjph.2017.04.021’,
 ’conditions_forDOI’: [{’act_id’: 4,
                    ’act_token’: ’calcinated’,
                    ’act_type’: ’Heating’,
                    ’env_ids’: [[42], []],
                    ’env_toks’: [’BaTiO3’, ’’],
                    ’ref_act’: False,
                    ’subject’: ’The Mixed powder’,
                    ’subsent’: [4, 21],
                    ’temp_values’: [{’max’: 800.0,
                                     ’min’: 800.0,
                                     ’tok_ids’: [15],
                                     ’units’: ’°C’,
                                     ’values’: [800.0]}],
                    ’time_values’: [{’max’: 2.0,
                                     ’min’: 2.0,
                                     ’tok_ids’: [9],
                                     ’units’: ’h’,
                                     ’values’: [2.0]}]},
                    ...
                  ],
 ’impurity_phase’: [{’additives’: [],
                         ’amounts_vars’: {},
                         ’composition’: [{’amount’: ’1’,
                                          ’elements’: {’Bi’: ’2’,
                                                       ’Fe’: ’4’,
                                                       ’O’: ’9’},
                                          ’formula’: ’Bi2Fe4O9’,
                                          ’species’: {’Bi’: ’2’,
                                                      ’Fe’: ’4’,
                                                      ’O’: ’9’}}],
                         ’elements_vars’: {},
                         ’is_acronym’: False,
                         ’material_formula’: ’Bi2Fe4O9’,
                         ’material_name’: ’’,
                         ’material_string’: ’Bi2Fe4O9’,
                         ’mp_id’: ’mp-504615’,
                         ’oxygen_deficiency’: None,
                         ’phase’: ’’},
                        {’additives’: [],
                         ’amounts_vars’: {},
                         ’composition’: [{’amount’: ’1’,
                                          ’elements’: {’Bi’: ’25’,
                                                       ’Fe’: ’1’,
                                                       ’O’: ’40’},
                                          ’formula’: ’Bi25FeO40’,
                                          ’species’: {’Bi’: ’25’,
                                                      ’FeO4’: ’0’}}],
                         ’elements_vars’: {},
                         ’is_acronym’: False,
                         ’material_formula’: ’Bi25FeO40’,
                         ’material_name’: ’’,
                         ’material_string’: ’Bi25FeO40’,
                         ’mp_id’: ’mp-759730’,
                         ’oxygen_deficiency’: None,
                         ’phase’: ’’}],
 ’impurity_reaction’: [{’left’: {’Bi2O3’: ’1’, ’Fe2O3’: ’2’},
                        ’right’: {’Bi2Fe4O9’: ’1’}},
                       {’left’: {’Bi2O3’: ’12.5’, ’Fe2O3’: ’0.5’, ’O2’: ’0.5’},
                        ’right’: {’Bi25FeO40’: ’1’}}],
 ’precursors’: [{’additives’: [],
                 ’amounts_vars’: {},
                 ’composition’: [{’amount’: ’1’,
                                  ’elements’: {’Bi’: ’2’, ’O’: ’3’},
                                  ’formula’: ’Bi2O3’,
                                  ’species’: {’Bi’: ’2’, ’O’: ’3’}}],
                 ’elements_vars’: {},
                 ’is_acronym’: False,
                 ’material_formula’: ’Bi2O3’,
                 ’material_name’: ’’,
                 ’material_string’: ’Bi2O3’,
                 ’mp_id’: ’mp-23501’,
                 ’oxygen_deficiency’: None,
                 ’phase’: ’’},
                {’additives’: [],
                 ’amounts_vars’: {},
                 ’composition’: [{’amount’: ’1’,
                                  ’elements’: {’Fe’: ’2’, ’O’: ’3’},
                                  ’formula’: ’Fe2O3’,
                                  ’species’: {’Fe’: ’2’, ’O’: ’3’}}],
                 ’elements_vars’: {},
                 ’is_acronym’: False,
                 ’material_formula’: ’Fe2O3’,
                 ’material_name’: ’’,
                 ’material_string’: ’Fe2O3’,
                 ’mp_id’: ’mp-23501’,
                 ’oxygen_deficiency’: None,
                 ’phase’: ’’}],
 ’target’: [{’additives’: [],
             ’amounts_vars’: {},
             ’composition’: [{’amount’: ’1’,
                              ’elements’: {’Bi’: ’1’, ’Fe’: ’1’, ’O’: ’3’},
                              ’formula’: ’BiFeO3’,
                              ’species’: {’Bi’: ’1’, ’FeO3’: ’1’}}],
             ’elements_vars’: {},
             ’is_acronym’: False,
             ’material_formula’: ’BiFeO3’,
             ’material_name’: ’’,
             ’material_string’: ’BiFeO3’,
             ’mp_id’: ’mp-23501’,
             ’oxygen_deficiency’: None,
             ’phase’: ’’}],
 ’target_reaction’: [{’left’: {’Bi2O3’: ’0.5’, ’Fe2O3’: ’0.5’},
                      ’right’: {’BiFeO3’: ’1’}}]}
```

4 Reactions that appear more in phase-pure dataset

$Fe_2O_3 + 0.8NiO + 0.2ZnO \longrightarrow Ni_{0.8}Zn_{0.2}Fe_2O_4$	1.0 (28 / 28)
$0.3CaCO_3 + 0.35La_2O_3 + MnO_2 \longrightarrow La_{0.7}Ca_{0.3}MnO_3 + 0.3CO_2 + 0.175O_2$	1.0 (26 / 26)
$BaCO_3 + CuO \longrightarrow BaCuO_2 + CO_2$	1.0 (22 / 22)
$0.4Bi + 1.6Sb + 3Te \longrightarrow Bi_{0.4}Sb_{1.6}Te_3$	0.971 (33 / 34)
$0.35La_2O_3 + MnO_2 + 0.3SrCO_3 \longrightarrow La_{0.7}Sr_{0.3}MnO_3 + 0.3CO_2 + 0.175O_2$	0.964 (27 / 28)
$La_2O_3 + 2TiO_2 \longrightarrow La_2Ti_2O_7$	0.964 (27 / 28)
$0.5In_2O_3 + 0.5Nb_2O_5 \longrightarrow InNbO_4$	0.963 (26 / 27)
$CoO + Fe_2O_3 \longrightarrow CoFe_2O_4$	0.963 (26 / 27)
$Ti_2AlC + TiC \longrightarrow Ti_3AlC_2$	0.941 (32 / 34)
$0.5Fe_2O_3 + 0.5Nb_2O_5 \longrightarrow FeNbO_4$	0.935 (29 / 31)
$Na_2CO_3 + 3TiO_2 \longrightarrow Na_2Ti_3O_7 + CO_2$	0.929 (26 / 28)
$0.5Cr_2O_3 + 0.5La_2O_3 \longrightarrow LaCrO_3$	0.923 (24 / 26)
$CaCO_3 + WO_3 \longrightarrow CaWO_4 + CO_2$	0.923 (24 / 26)
$2K_2CO_3 + 3Nb_2O_5 \longrightarrow K_4Nb_6O_{17} + 2CO_2$	0.92 (23 / 25)
$Li_2CO_3 + 3TiO_2 + ZnO \longrightarrow Li_2ZnTi_3O_8 + CO_2$	0.92 (23 / 25)
$0.333Co_3O_4 + Fe_2O_3 \longrightarrow CoFe_2O_4 + 0.167O_2$	0.917 (44 / 48)
$Pb + Te \longrightarrow PbTe$	0.914 (32 / 35)
$BaCO_3 + 0.8TiO_2 + 0.2ZrO_2 \longrightarrow BaZr_{0.2}Ti_{0.8}O_3 + CO_2$	0.913 (21 / 23)
$2Bi_2O_3 + 3TiO_2 \longrightarrow Bi_4Ti_3O_{12}$	0.912 (114 / 125)

Table S1. Reactions appearing more in phase-pure dataset. Reactions with a phase-pure count larger than 20 with a phase-pure/total ratio larger than 0.91 are shown.

5 Reactions that appear more in phase-impure dataset

Reactions	Fraction pure	Impurity phases
$C + SiC + 3Ti \longrightarrow Ti_3SiC_2$	0.185 (5 / 27)	TiC(6)<br>$Ti_5Si_3Cx + TiCx$(3)<br>unspecified (3)
$Si + Ti + 2TiC \longrightarrow Ti_3SiC_2$	0.324 (11 / 34)	unspecified (7)<br>$Ti_5Si_3 + TiC$(6)<br>TiC(4)
$2C + Si + 3Ti \longrightarrow Ti_3SiC_2$	0.366 (26 / 71)	TiC(16)<br>$SiC + TiC$(6)<br>unspecified (6)<br>$TiC + TiSi_2$(4)<br>$Ti_5Si_3 + TiC$(3)
$Al + C + 2Cr \longrightarrow Cr_2AlC$	0.368 (14 / 38)	unspecified (6)<br>Cr_7C_3(5)<br>$Cr_2Al + Cr_7C_3$(4)<br>$Al_2O_3 + Cr_7C_3$(3)
$Ni + Ti \longrightarrow NiTi$	0.417 (15 / 36)	$Ni_4Ti_3 + Ti_2Ni$(3)
$3Sb + 4Zn \longrightarrow Zn_4Sb_3$	0.447 (21 / 47)	$ZnSb$(7)<br>$Zn + ZnSb$(5)<br>unspecified (3)
$0.5Bi_2O_3 + 0.5Fe_2O_3 \longrightarrow BiFeO_3$	0.484 (107 / 221)	$Bi_{25}FeO_{40} + Bi_2Fe_4O_9$(18)<br>unspecified (18)<br>$Bi_2Fe_4O_9$(17)<br>$Bi_{25}FeO_{39} + Bi_2Fe_4O_9$(16)<br>$Bi_{25}FeO_{40}$(6)<br>$Bi_2Fe_4O_9 + Bi_2O_3$(4)<br>$Bi_2O_3$(3)
$Mo + 2Si \longrightarrow MoSi_2$	0.488 (20 / 41)	Mo_5Si_3(9)
$Al + 2C + 3Ti \longrightarrow Ti_3AlC_2$	0.571 (52 / 91)	TiC(14)<br>unspecified (9)<br>$Ti_2AlC + TiC$(4)

Table S2. Reactions appearing more in phase-impure dataset. Reactions with a phase-impure count larger than 20 with a ratio of phase-impure/total smaller than 0.58 are shown.

6 Dependence of outcome on precursors for Ti_3AlC_2 target

Reaction	Fraction pure	Impurity phases
$Al + 2C + 3Ti \longrightarrow Ti_3AlC_2$	0.571 (52 / 91)	TiC(14)<br>unspecified (9)<br>$Ti_2AlC + TiC$(4)
$Al + Ti + 2TiC \longrightarrow Ti_3AlC_2$	0.78 (46 / 59)	$Ti_2AlC + TiC$(3)<br>TiC(3)
$Ti_2AlC + TiC \longrightarrow Ti_3AlC_2$	0.941 (32 / 34)	TiC(2)

Table S3. Synthesis reactions for Ti_3AlC_2 and total count larger than 30.

7 Unreacted precursors

Reactions	Fraction of unreacted precursors	Unreacted precursors
$Al + Ti + 2TiC \longrightarrow Ti_3AlC_2$	0.923 (12 / 13)	TiC(11)<br>$Ti + TiC$(1)
$CaCO_3 + 3CuO + 4TiO_2 \longrightarrow CaCu_3Ti_4O_{12} + CO_2$	0.667 (40 / 60)	CuO(20)<br>TiO_2(10)<br>$CuO + TiO_2$(8)
$Al + 1.5H_2O + NaH \longrightarrow NaAlH_4$	0.615 (8 / 13)	Al(8)
$Si + Ti + 2TiC \longrightarrow Ti_3SiC_2$	0.609 (14 / 23)	TiC(14)
$3Sb + 4Zn \longrightarrow Zn_4Sb_3$	0.538 (14 / 26)	Zn(10)
$TiO_2 + ZnO \longrightarrow ZnTiO_3$	0.538 (7 / 13)	TiO_2(6)
$3CaCO_3 + 1.333Co_3O_4 + 0.333O_2 \longrightarrow Ca_3Co_4O_9 + 3CO_2$	0.5 (7 / 14)	Co_3O_4(7)
$2Li_2CO_3 + 5TiO_2 \longrightarrow Li_4Ti_5O_{12} + 2CO_2$	0.483 (14 / 29)	TiO_2(14)
$Li_2CO_3 + 2SiO_2 \longrightarrow Li_2Si_2O_5 + CO_2$	0.462 (6 / 13)	SiO_2(5)
$Co + 3Sb \longrightarrow CoSb_3$	0.462 (6 / 13)	Sb(6)
$C + Sn + 2Ti \longrightarrow Ti_2SnC$	0.455 (5 / 11)	Sn(5)
$2Mg + Ni \longrightarrow Mg_2Ni$	0.444 (8 / 18)	$Mg + Ni$(4)<br>Ni(3)
$Bi + Mn \longrightarrow MnBi$	0.412 (7 / 17)	Bi(4)<br>$Bi + Mn$(3)
$BaCO_3 + 6Fe_2O_3 \longrightarrow BaFe_{12}O_{19} + CO_2$	0.391 (9 / 23)	Fe_2O_3(8)

Table S4. Reactions that are more likely to have unreacted precursors. The second column shows the fraction of reactions with unreacted precursors in the phase-impure dataset. Reactions with phase-impure count larger than 10 and with such fraction larger than 0.39 are shown.

Precursors	Fraction of reactions with precursor in impurity phases	Targets
TiC	0.594 (38 / 64)	Ti_3SiC_2(31)<br>Ti_3AlC_2(18)<br>Ti_2AlC(9)
HfO_2	0.452 (19 / 42)	$SrHfO_3$(4)<br>$Ca_2LuHf_2Al_3O_{12}$(3)<br>$Hf_6Ta_2O_{17}$(3)<br>$La_2Hf_2O_7$(3)<br>$BaHfSi_3O_9$(2)<br>HfW_2O_8(2)<br>$Eu_2Hf_2O_7$(2)
SrF_2	0.417 (10 / 24)	Sr_3AlO_4F(4)<br>Sr_2FeO_3F(2)<br>$LiSrAlF_6$(2)
Ga_2S_3	0.385 (5 / 13)	$CaGa_2S_4$(3)
ZnS	0.367 (11 / 30)	Cu_2ZnSnS_4(14)<br>CaZnOS(13)
⋮		
$CaCO_3$	0.008 (6 / 731)	$CaCu_3Ti_4O_{12}$(61)
$MnCO_3$	0.005 (1 / 186)	$LiNi_{0.5}Mn_{1.5}O_4$(11)
H_3BO_3	0.0 (0 / 255)	$SrAl_2O_4$(14)
$NH_4H_2PO_4$	0.0 (0 / 231)	$Li_{1.5}Al_{0.5}Ge_{1.5}(PO_4)_3$(15)
$(NH_4)_2HPO_4$	0.0 (0 / 67)	$LiFePO_4$(5)
$MgCO_3$	0.0 (0 / 56)	Mg_2SiO_4(8)<br>$MgAl_2O_4$(8)

Table S5. Precursors that are more/less likely to be observed as final impurity phase. The second column shows the fraction of reactions with a specific precursor in impurity phases out of the total count of reactions when the precursor is used in the phase-impure dataset. In the above box, Precursors with a frequency in the phase-impure dataset larger than 10 and with such fraction larger than 0.3 are shown, and the below box shows compound precursors with a frequency in the phase-impure dataset larger than 50 and with a fraction lower than 0.01. The most common targets of the reactions involving a precursor in the phase-impure dataset are shown in the third column.

8 Paragraph Classification and Manual Validation

All paragraphs were subjected to a section heading filter using the regular expression:

```
query = {"path": {"$regex": "preparation|prepared|experiment|method|material|synthes", "
    $options": "i"}}
```

This allowed to apply MatBERT only to paragraphs potentially relevant to synthesis. Then, we applied MatBERT to the rest of the paragraphs with batch size of 16.

Since our dataset is unbalanced by publisher and class labels, we sampled 10 paragraphs for each of 8 publishers and 6 class labels. Then, human annotators manually validated class labels for all 480 paragraphs and discussed them. Below are guidelines for manual annotation.

8.1 Solid-State

Synthesis from powders via mechanical mixing/grinding and subsequent heating steps. Regrinding and reheating steps are common. Typically starting from oxide precursors. Can be used for other morphologies, like thin films (via pulsed laser deposition (PLD) or sputtering).

Common keywords: solid-state method, solid-state reaction, ball-milling.

Process: Involves mixing powders of precursor materials, followed by mechanical grinding and high-temperature heating (calcination and sintering). Key Steps: Mixing and grinding precursor powders. Heating to high temperatures to induce solid-state reactions. Regrinding and reheating if necessary. Applications: Widely used for creating ceramics, oxides, and other crystalline materials. Example: Mixing Ni powder and g-C3N4, ball milling, vacuum drying, and sintering.

8.2 Sol-Gel

Synthesis from hydrated precursors via chemical dissolution. Includes solvents, chelating agents, and other possible additives in addition to reaction precursors. Can be used for additional morphologies, like thin films (via spin coating or drop casting).

Common keywords: sol-gel method, Nitrate or acetate precursors, use of acidic chelating agents, pechini method.

Process: Involves hydrolysis and polymerization of metal alkoxides or other precursors in a solution to form a colloidal suspension (sol) that gels to form a network (gel). Key Steps: Dissolving precursors in a solvent. Hydrolysis and condensation reactions to form a sol. Gelation to form a gel. Drying and heating to remove solvents and form the final material. Applications: Used for making glasses, ceramics, thin films, and coatings. Example: Dissolving Ni(NO3)2 and Co(NO3)2 in ethanol, stirring, drying, and calcining.

8.3 Hydrothermal

Synthesis from aqueous solution carried out in an autoclave, typically at high pressure which allows for lower temperature (<300C) to crystallize target.

Common keywords: hydrothermal, autoclave.

Process: Uses high-temperature aqueous solutions in an autoclave to crystallize materials from a solution at high vapor pressures. Key Steps: Dissolving precursors in water. Transferring the solution to an autoclave. Heating the autoclave to promote crystal growth. Cooling, filtering, and washing the product. Applications: Commonly used for synthesizing nanomaterials, zeolites, and single crystals. Example: Dissolving sodium tungstate and oxalic acid, transferring to an autoclave, heating at 180°C, and cooling to obtain WO3 nanowires.

8.4 Precipitation

Synthesis from mixing of precursors in aqueous solution followed by filtering (to obtain the precipitated product) and possible subsequent heating.

Common keywords: precipitation, coprecipitation, filtered, ultrasonication.

Process: Involves mixing aqueous solutions of metal salts and a precipitating agent to form insoluble solid particles that are then collected and processed. Key Steps: Dissolving metal salts in water. Adding a precipitating agent to form a precipitate. Filtering and washing the precipitate. Drying and sometimes calcining the precipitate. Applications: Used for making various inorganic compounds, including oxides, hydroxides, and carbonates. Example: Mixing BaCl2 and NH3F in water, filtering, washing, drying, and annealing to obtain BaF2 nanoparticles.

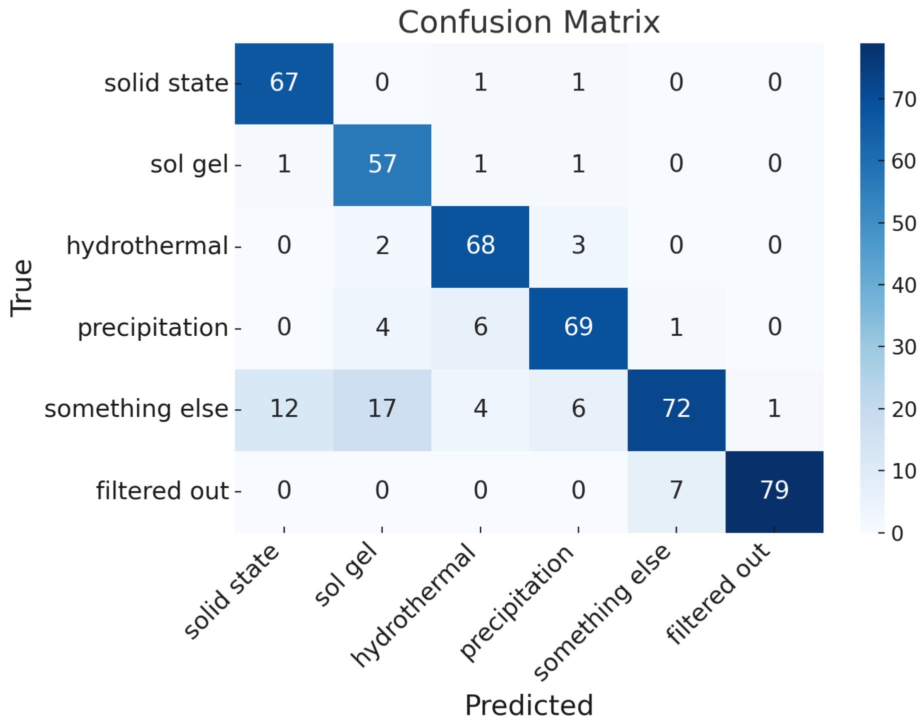

Figure S1. Confusion matrix comparing the ground truth with the classifier predictions.

Reaction energies for impurity phase formation

To potentially gain insights beyond E_{hull}, we analyzed the reaction energies required to form either the target or the impurity phase from the same precursors. Szymanski et al. recently demonstrated that if one phase in a solid-state reaction has a driving force of at least 60meV/atom greater than all competing phases, it is predicted to form as an initial product. Conversely, when multiple phases share comparable driving forces, the initial product is more often governed by kinetic factors[6]. We tested if we could extend this principle to the final phase purity by comparing the reaction energies for forming the target versus an impurity and assessing whether their difference correlates with the observed presence or absence of impurity phases.

Next, we further narrowed this set to focus on 838 reactions for which we could reliably compute reaction energies. In particular, this requires the synthesis reaction to be fully balanced not only for the target but also for any extracted impurity phase. Furthermore, we excluded reactions involving gas phases (CO_2, O_2, H_2O, NH_3, NO_2), because their computed formation energies in the Materials Project do not accurately reflect the gas phase. We then computed reaction energies for target and impurity phase reactions using the `ComputedReaction` class from pymatgen software[7].

Figure S2 compares the computed reaction energies at 300K for forming either the target or impurity phase from the same precursors. These computed reaction energies reflect initial interfacial reactions between precursor powders, which may indicate the relative likelihood of target versus impurity phase formation in the early stages of synthesis. On average, the impurity-forming reaction energies were approximately 20meV/atom more negative than those forming the target, with 36% favoring the impurity phase. Thus, as expected, the phase-impure reactions also had a greater thermodynamic driving force favoring impurity phase formation over the desired target. However, 21% of the phase-impure cases favor the target thermodynamically yet still yield impurity phases, indicating that kinetic factors, reaction pathways, or other intermediate phases must also be influencing these outcomes. In summary, while thermodynamic descriptors like E_{hull} and reaction energies offer valuable initial screening, our findings demonstrate the need to more directly model process conditions and kinetic barriers to fully understand and control impurity formation in solid-state syntheses.

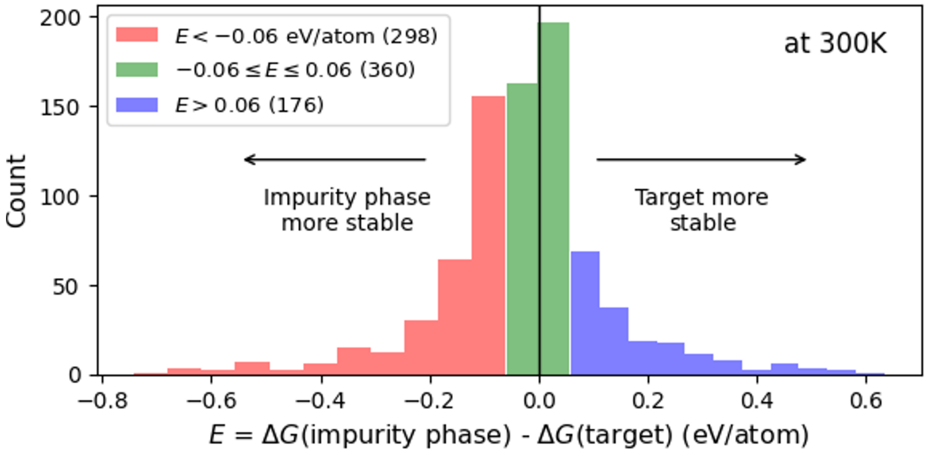

Figure S2. Histogram of difference in reaction energies from precursors to form either a target or impurity phase. Target and impurity phase material formulas were queried to Materials Project to fetch energy above hull. Four outliers were manually checked and filtered out.

References

1. Lee, K. *et al.* Selection of binder and solvent for solution-processed all-solid-state battery. *J. The Electrochem. Soc.* **164**, A2075, 10.1149/2.1341709jes (2017).

2. Le, T. *et al.* The electronic structure of the curh1xmgxo2 thermoelectric materials: An x-ray photoelectron spectroscopy study. *J. Solid State Chem.* **184**, 2387–2392, https://doi.org/10.1016/j.jssc.2011.07.005 (2011).

3. Johnston, D. C. *et al.* Magnetic exchange interactions in bamn$_2$as$_2$: A case study of the J_1-J_2-J_c heisenberg model. *Phys. Rev. B* **84**, 094445, 10.1103/PhysRevB.84.094445 (2011).

4. Qwen *et al.* Qwen2.5 technical report (2025). 2412.15115.

5. Kononova, O. *et al.* Text-mined dataset of inorganic materials synthesis recipes. *Sci. Data* **6**, 10.1038/s41597-019-0224-1 (2019).

6. Szymanski, N. J. *et al.* Quantifying the regime of thermodynamic control for solid-state reactions during ternary metal oxide synthesis. *Sci. Adv.* **10**, eadp3309, 10.1126/sciadv.adp3309 (2024). https://www.science.org/doi/pdf/10.1126/sciadv.adp3309.

7. Ong, S. P. *et al.* Python materials genomics (pymatgen): A robust, open-source python library for materials analysis. *Comput. Mater. Sci.* **68**, 314–319, https://doi.org/10.1016/j.commatsci.2012.10.028 (2013).
